# Moisture Sorption Isotherms of Polydextrose and Its Gelling Efficiency in Inhibiting the Retrogradation of Rice Starch

**DOI:** 10.3390/gels10080529

**Published:** 2024-08-12

**Authors:** Chang Liu, Xiaoyu Li, Hongdong Song, Xingjun Li

**Affiliations:** 1 National Engineering Research Center for Grain Storage and Transportation, Academy of National Food and Strategic Reserves Administration, Beijing 102209, China; shlglc@126.com; 2 College of Health Science and Engineering, University of Shanghai for Science and Technology, Shanghai 200093, China; cau4080@163.com; 3 Department of Pharmacy, Sichuan University, Chengdu 610041, China

**Keywords:** polydextrose, desorption isotherms, sorption hysteresis, polynomial, gelling agent, starch retrogradation

## Abstract

As an anti-staling agent in bread, the desorption isotherm of polydextrose has not been studied due to a very long equilibrium time. The adsorption and desorption isotherms of five Chinese polydextrose products were measured in the range of 0.1–0.9 a_w_ and 20–35 °C by a dynamic moisture sorption analyzer. The results show that the shape of adsorption and desorption isotherms was similar to that of amorphous lactose. In the range of 0.1–0.8 a_w_, the hysteresis between desorption and adsorption of polydextrose was significant. The sorption isotherms of polydextrose can be fitted by seven commonly used models, and our developed seven-parameter polynomial, the adsorption equations of generalized D’Arcy and Watt (GDW) and Ferro-Fontan, and desorption equations of polynomial and Peleg, performed well in the range of 0.1–0.9 a_w_. The hysteresis curves of polydextrose at four temperatures quickly decreased with a_w_ increase at a_w_ ˂ 0.5, andthereafter slowly decreased when a_w_ ≥ 0.5. The polynomial fitting hysteresis curves of polydextrose were divided into three regions: ˂0.2, 0.2–0.7, and 0.71–0.9 a_w_. The addition of 0–10% polydextrose to rice starch decreased the surface adsorption and bulk absorption during the adsorption and desorption of rice starch, while it increased the water adsorption value at a_w_ ≥ 0.7 due to polydextrose dissolution. DSC analysis showed that polydextrose as a gelling agent inhibited the retrogradation of rice starch, which could be used to maintain the quality of cooked rice.

## 1. Introduction

Hydrocolloids are colloidal substances with an affinity for water, and they produce viscous solutions, pseudo-gels, or gels in water [1]. Polydextrose is a high molecular weight polysaccharide that can provide viscosity in the food industry [2]. Polydextrose can be synthesized from D-glucose, sorbitol, and citric acid with a proportion of 89:10:1 in a reaction kettle through high-temperature melting and a vacuum concentration process [3]. The production of synthetic polydextrose in China was 101,000 tons in 2020 with an annual output value of USD 0.143 billion, and expected output will be 135,000 tons in 2026 [4]. This was used in health products, beverages, fermented dairy products, and baked products (39.0%, 21.6%, 16.6%, and 11.8%, respectively) and chocolates, nutrition bars, and other foods (11.0%). Polydextrose is a randomly linked glucose polymer by all possible glycosidic linkages, including α- and β-1–2, 1–3, 1–4, and 1–6, with some branching [2]. It has an average degree of polymerization (DP) of 12. It is an amorphous powder that is weakly acidic, hygroscopic, and water-soluble. As a soluble dietary fiber, synthetic polydextrose is beneficial to human health, although it is costly [5]. Polydextrose has bulking properties with a low sweetness level of only 10% of the sweetness of sucrose [6]. It provides energy of 1 kcal/g and has a 90 g/day consumption threshold because excessive consumption of polydextrose might cause a laxative effect [7]. However, few studies have dealt with the desorption and adsorption isotherms of polydextrose.

Moisture sorption characteristics of biological materials play very important roles in technological processes such as drying, handling, mixing and packaging, storage, and other processes that require the estimation of drying time, prediction of food stability, shelf life, glass transition and texture, and prevention of deteriorative reactions [8]. The understanding of a moisture sorption isotherm model and hysteresis assessment procedures will be useful in development, selection, modeling, and controlling as well as making optimization of appropriate processes for enhanced efficiency in the food processing value chain [9,10]. The commonly used equilibrium moisture content (EMC) models for biological materials, namely Brunauer–Emmett–Teller (BET), Guggenheim–Anderson–deBoer (GAB), modified GAB (MGAB), and modified Hailwood–Horrobin (MHH), as well as modified Halsey (MHE), modified Henderson (MHE), modified Chung–Pfost (MCPE), and modified Oswin (MOE), have been evaluated for different foods, but with the gradual advance in the determination technique of moisture sorption isotherms in recent years [8,11], new or modified equations are still necessary.

**Scheme 1:**
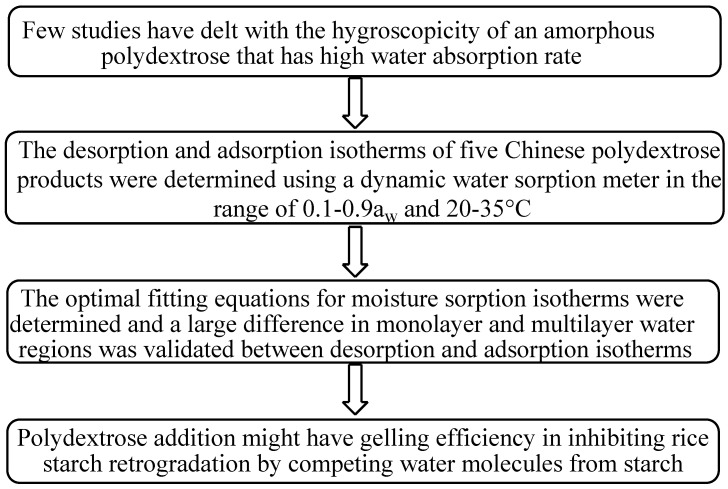
Schematic diagram of the present study.

Polysaccharides such ascarboxymethyl cellulose (CMC),crude tea polysaccharide,β-glucan, guar gum, and gum arabic (GA), as well as iota-carrageenan, konjac glucomannan, soybean-soluble polysaccharide (SSPS), pectin, and xanthan gum, are used as additives to retard starch retrogradation [12]. The mechanisms for the inhibition of starch retrogradation would be explained in terms of competitionfor water between starch and thesepolymers [13]. The primary effect of polydextrose in reducing the staling rate in baked products is thought to be due to its higher water absorption capacity and diluting of the starch components, thereby reducing the available starch fractions for crystallization [14]. The objective is to investigate the hygroscopic behavior of Chinese polydextrose products and to apply mathematical models to predict their adsorption and desorption isotherms obtained by a dynamic water sorption analyzer (Scheme 1), with an aim to elucidate the mechanism of their moisture retention and gelling efficiency when addedto food.

## 2. Results and Discussion

### 2.1. Experimental EMC/ERH Data for Polydextrose Samples

**Figure 1:**
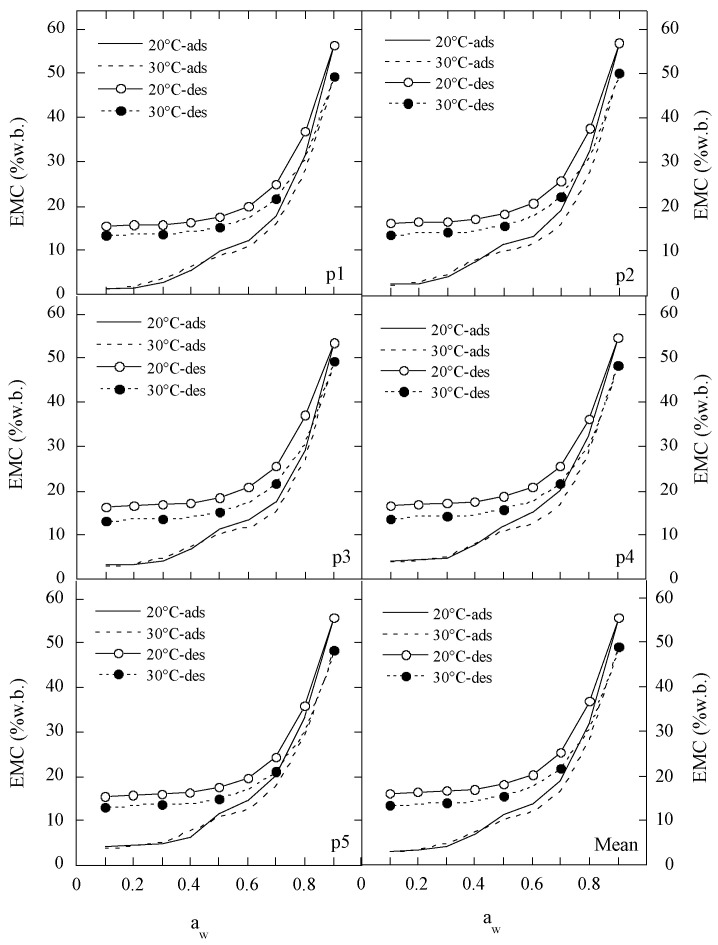
The measure moisture isotherms of polydextrose. Note: EMC, equilibrium moisture content; w.b., wet basis; a_w_, water activity; ads, adsorption; des, desorption; p1, p2, p3, p4, and p5 are five polydextrose samples; Mean is the average isotherms of five samples.

At water activity ranging from 0.1 to 0.9 and four temperatures (20, 25, 30, and 35 °C), the adsorption and desorption isotherms of five polydextrose samples were measured and are shown for the 20 and 30 °C isotherms in Figure 1. Both adsorption and desorption isotherms were sigmoidal in shape. At the same temperature, there were significantdifferences between adsorption and desorption isotherms in the range of 0.1 to 0.7 *a*_w_. With an increase in the initial moisture content (MC) of samples, the isotherms at the same temperature were slightly raised for desorption or adsorption. At 0.3–0.7 *a*_w_, there was a significant inflexion point in the adsorption isotherm for five samples, indicating the surface adsorption and bulk absorption.

### 2.2. Fitting of Sorption Equations to Experimental Sorption Data

The statistical parameters used to compare the model fits (*R*^2^, *RSS*, *SE*, and *MRE*) are given in Table 1, Table 2 and Table 3. The eight equations, namely Ferro-Fontan, the generalized D’Arcy and Watt (GDW), Boquet, Lewicki, Iglesias, MGAB, Peleg, and our developed polynomial, all can provide good fits to the experimental sorption isotherm data of polydextrose samples in the range of 0.1–0.9 *a*_w_, although Iglesias, MGAB, and polynomial equations gave larger *MRE* values for adsorptive EMC data.

Further comparisons of the sorption equations were made for five sets of polydextrose isotherm data (Table 4), and average values of the *R*^2^ and error parameters (*RSS*, *SE* and *MRE*) were calculated. For the adsorption, the equations were ranked in the following order of accuracy from the highest to the lowest: GDW, Ferro-Fontan, Peleg, MGAB, Boquet, Lewicki, polynomial, and Iglesias. In the case of desorption, the order was polynomial, Peleg, Iglesias, GDW, Boquet, Lewicki, Ferro-Fontan, and MGAB. The adsorption equations of GDW and Ferro-Fontan and the desorption equations of polynomial and Peleg well described the equilibrium moisture data of the five species of polydextrose in the range of 0.1–0.9 *a*_w_. The coefficients of the best-fitting equations are summarized in Table 3 and Table 5. These calculated coefficients can be used to describe the process of polydextrose dehydration and improve the physical control of moisture during package and storage.

### 2.3. Prediction of Moisture Sorption Isotherms by the Best Fitting Equation

**Figure 2:**
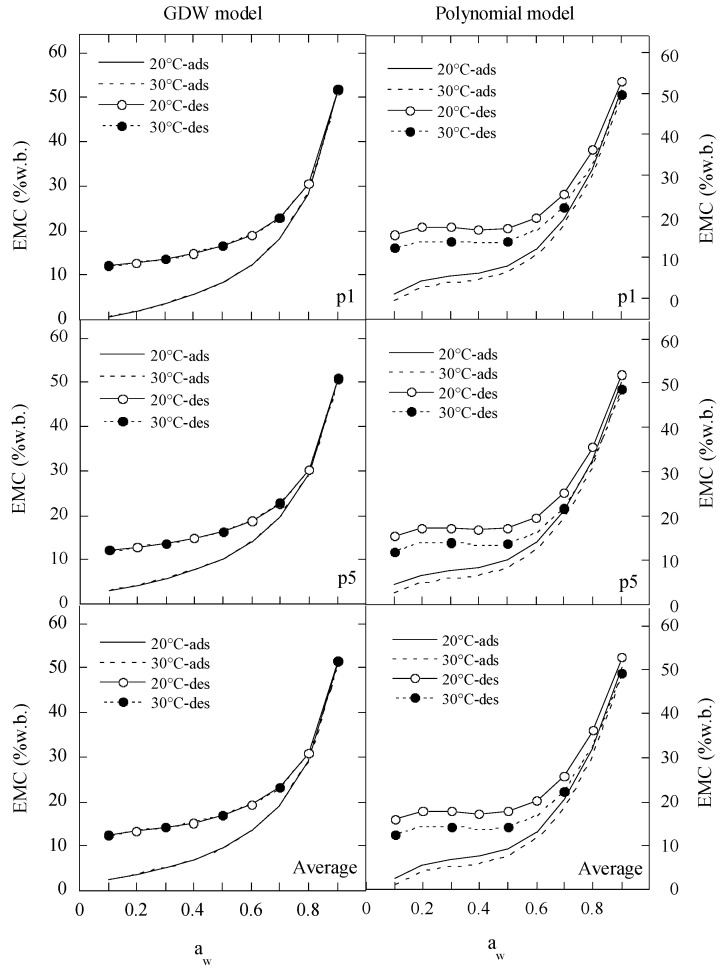
The predicted adsorption and desorption isotherms of polydextrose samples. Note: EMC, equilibrium moisture content; w.b., wet basis; a_w_, water activity; ads, adsorption; des, desorption; GDW, generalized D’Arcy and Watt; p1 and p5 are the samples; Average is the mean of adsorption or desorption isotherms for five samples.

The predicted sorption isotherms of a1 and a5 polydextrose samples at 20 and 30 °C are displayed in Figure 2. At *a*_w_≤ 0.8, the hysteresis between adsorption and desorption becomes bigger with a decrease in a_w_ for two temperatures. At *a*_w_ ≤ 0.6, the adsorption isotherms of the p5 sample were higher than those of the p1 sample. These results suggest that the difference might occur in the properties of monolayer and multilayer water sorption.

### 2.4. The Hysteresis Degree between Moisture Desorption and Adsorption of Polydextrose

**Figure 3:**
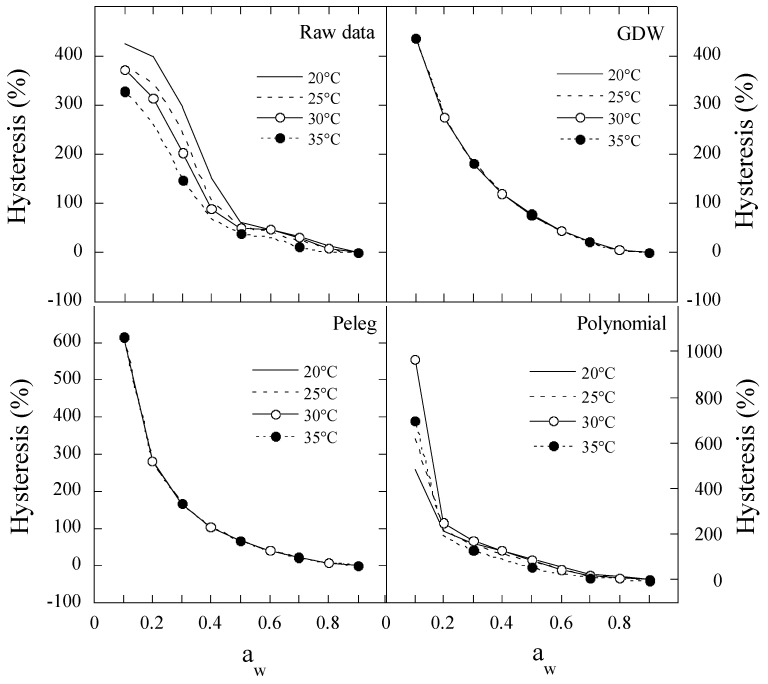
The hysteresis degree of moisture sorption in polydextrose. Note: a_w_, water activity; GDW, generalized D’Arcy and Watt.

The raw hysteresis degree curve of moisture sorption in polydextrose decreased sharply with an increase in water activity below 0.5 *a*_w_, and then decreased slowly with increasing water activity. With an increase in temperature, hysteresis degree curve moved down (Figure 3). The fitted hysteresis degree curves by the GDW and Peleg equations show hyperbolas and could not distinguish the effect of temperature, but hysteresis degree curves fitted by the polynomial can show the effect of temperature. The hysteresis degree curves fitted by the polynomial can be divided into three stages at ˂ 0.2 *a*_w_, 0.2–0.7 *a*_w_, and 0.71–0.9 *a*_w_, corresponding tothe hysteresis degree decreasing quickly, slowly, and flatly, respectively.

### 2.5. Change in the Phase State of Rice Starch with Polydextrose Addition

**Figure 4:**
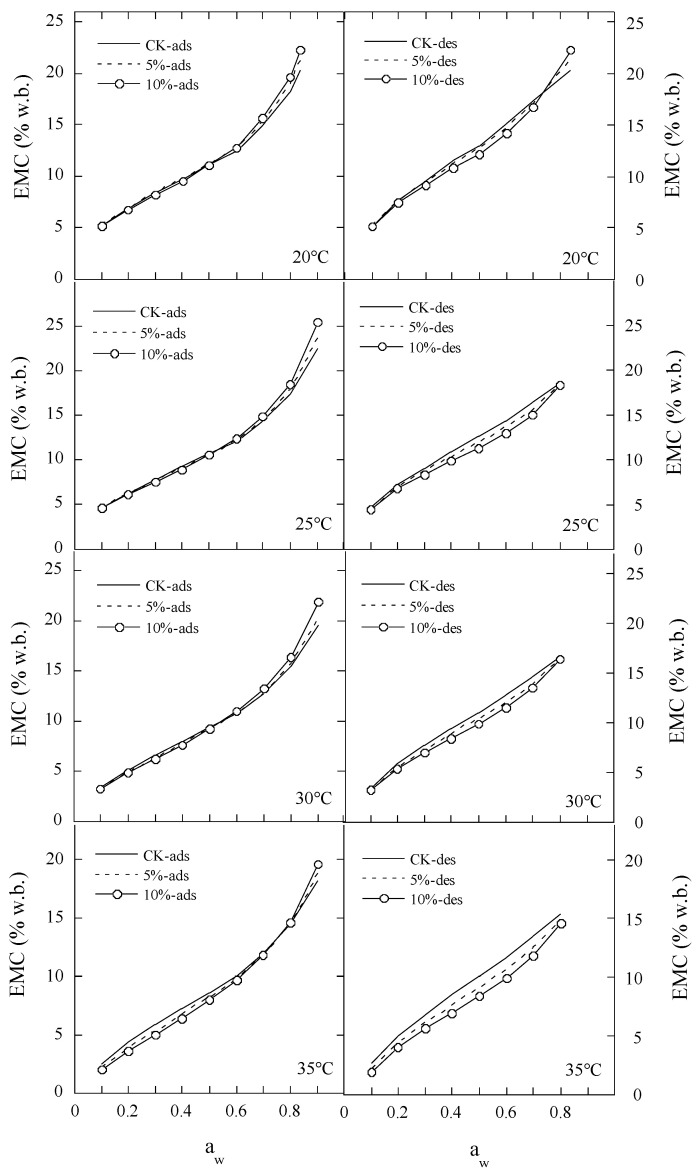
Effect of polydextrose addition on the moisture sorption isotherms of rice starch.Note: EMC, equilibrium moisture content; w.b., wet basis; a_w_, water activity; CK, the control rice starch; ads, adsorption; des, desorption.

For the moisture adsorption isotherms of rice starch at 20, 25, 30, and 35 °C, amorphous polydextrose addition can increase water adsorptive values after about 0.7 *a*_w_ due to the dissolution of the polydextrose (Figure 4). With an increase in temperature, polydextrose addition decreased the surface adsorption and bulk absorption in the range of 0.2 to 0.65 *a*_w_. For the desorption isotherms of rice starch at 20–35 °C, polydextrose addition decreased the moisture desorption values in the range of 0.2 to 0.75 *a*_w_. These results suggest that polydextrose addition decreased the surface adsorption and bulk absorption during moisture adsorption and desorption of rice starch, but increased moisture sorption values after about 0.7 *a*_w_ due to the dissolution of the polydextrose.

Compared with the control, with an increase in polydextrose addition level from 3% to 10% in rice starch, the *T*_o_, *T*_p_, peak width, peak height of gelatinization had almost no changes (Table 6), but gelatinization enthalpy and *T*_c_ significantly decreased, indicating that amorphous polydextrose addition could inhibit the starch retrogradation and aging and can be used to keep the quality of cooked rice.

There are few studies on the moisture adsorption isotherms of non-crystalline polydextrose. Wong et al. [2] measured the 29 °C isotherm of polydextrose adsorption in the range of 17–87% ERH using saturated salt solution and a static gravimetric technique. They figured out that polydextrose could be classified as a type 2 isotherm (sigmoid shape) because there was an inflexion point in the isotherm. The inflexion point at 0.6–0.75 *a*_w_ indicates the occurrence of a moisture-induced phase transition, i.e., glass transitions and successive crystallization in the samples [15]. In this study, at 0.3–0.7 *a*_w,_ there was a significant inflexion point in the adsorption isotherm for five polydextrose samples measured by a dynamic water sorption analyzer (SPS11−10μ), and indicated the surface adsorption and bulk absorption, which is the region of glassy to rubbery phase transition. Similarly, Li and Schmidt [16] determined the polydextrose isotherms in the range of 11.3–84.3% ERH and 20–40 °C using a dynamic vapor sorption (DVS) ramping and equilibrium method, and indicated the surface adsorption and bulk absorption in the range of 0.25–0.65 *a*_w_. We also confirmed the region of surface adsorption and bulk absorption with the hysteresis degree curves fitted by a polynomial equation that can be divided into three stages at ˂ 0.2 *a*_w_, 0.2–0.7 *a*_w_, and 0.71–0.9 *a*_w_.

In the present study, the moisture adsorption and desorption isotherm shapes of polydextrose were determined to be similar to those of lactose monohydrate [17]. There was a huge hysteresis between desorption and adsorption isotherm of polydextrose at the same temperature in the range of 0.1–0.8 *a*_w_. Li and Schmidt [16] used a four-parameter polynomial equation (Li–Schmidt equation) to fit the experimental adsorption data of polydextrose. In this study, to our knowledge, we are the first to simultaneously determine the adsorption and desorption isotherms of five Chinese polydextrose products, and then used eight equations to fit them, of which MGAB and polynomial contain temperature term, but the other six equations do not have temperature term.The adsorption equations of GDW and Ferro-Fontan, and the desorption equations of polynomial and Peleg were judged as well describing the equilibrium moisture data of the five species of polydextrose in the range of 0.1–0.9 *a*_w_. It is interesting that our developed seven-parameter polynomial could show the two transition points at 0.2 *a*_w_ and 0.7 *a*_w_, respectively, for surface adsorption and bulk absorption on the hysteresis degree curves.

When amorphous polydextrose was added to rice starch, the enthalpy and conclusion temperature (*T*_c_) of gelatinization significantly decreased, but the onset temperature (*T*_o_) and peak temperature (*T*_p_) of gelatinization were not significantly influenced. *T*_c_ reduction means the delay or inhibition of starch recrystallization or retrogradation [18]. We explain that, as a gelling agent, polydextrose could adsorb such a high amount of water as to dilute the starch components, thus reducing the available starch fractions for recrystallization. The decrease in surface adsorption and bulk absorption in rice starch by polydextrose addition suggests that the study on glass transition temperature and moisture sorption curves is interesting to elucidate the mechanism of preventing starch retrogradation by adding different polysaccharides or oligosaccharides.

## 3. Conclusions

The measurement time for the desorption isotherm of amorphous polydextrose was significantly longer than that of the adsorption isotherm at the same temperature, which may be due to the fact that the desorption from solution to surface takes place through the stage of surface desorption plusbulk desorption in the range of 0.7 to 0.3 *a*_w_, that is, from the rubber state to the glass state, and it showed that the hysteresis curve of polydextrose decreased rapidly with the increase of water activity in the range of 0.1 ˂ *a*_w_ ˂ 0.5, and then decreased slowly with the increase of water activity at *a*_w_ ≥ 0.5. When 3–10% polydextrose was added to rice starch, it was used to reduce the moisture desorption value of rice starch in the range of 0.3–0.7 *a*_w_, and significantly decreased the conclusion temperature of rice starch gelatinization and inhibited starch retrogradation. The addition of 3–10% polydextrose has application scenes in inhibiting starch aging in cereal foods.

## 4. Materials and Methods

### 4.1. Materials

High-purity nitrogen (99.999%)was purchased from Beiwen Gas Company, Beijing, China; sodium azide (analytical purity) was from GuoyaoJituan, Beijing, China. Rice starch was from Sigma-Aldrich (Shanghai, China) Trading Co., Ltd., Shanghai, China. A dynamic water sorption analyzer (SPS11−10μ) with SPS-Toolbox Basic Rel. 1.15 software was from ProUmid GmbH & Co. KG, Ulm, Germany. Differential scanning calorimeter (DSC, 200F3) was purchased fromNetzsch, Freistaat Bayern, Germany. The six polydextrose samples were collected from five plants in China (Table 7). The initial moisture contents of the samples were determined by the method of AOAC [19].

### 4.2. Moisture Sorption Isotherms and Their Fitting

Moisture sorption isotherms of the samples were determined with a dynamic water sorption analyzer. This instrument is an integrated system for automatic gravimetric determination of the water vapor adsorption and desorption of multiple samples in a test atmosphere with controlled temperature and water activity (a_w_). Temperature over time is ±0.1 K, and a_w_ accuracy is ±0.006 at (23 ± 5 °C) for the range of 0–1 a_w_. The sample turntable with 12 aluminum trays was used, and each tray has a bottom inner-diameter of 5.0 cm, top inner-diameter of 5.5 cm, outer-edge diameter of 6.3 cm and a height of 1.3 cm. The equilibrium moisture contents (EMCs) of each sample (ca. 2.0000 g) at four constant temperatures (20, 25, 30, and 35 °C) over a_w_ range of 0.1–0.9 were determined using deionized water producing humidity and high-purity nitrogen blowing dry and preventing samples from getting moldy. The interval time between gravimetric cycles was set up as 10 min. The measurement cycle was started at 0.1 a_w_ and first increased with a 0.1 step to 0.2 a_w_. Then, a_w_ was raised to 0.3, 0.4, 0.5, 0.6, 0.7, 0.8, and further to 0.9. The time per cycle was set to a minimum of 50 min and a maximum of 50 h. The default weight limit was +100%, and balance bandwidth (dm/dt) was ±0.01%/40 min. During the measurement cycle, the samples are automatically placed on an analytical balance and weighed. The sample pan remains unloaded and was used for drift compensation of the measured values. The recorded data were analyzed by SPS-Toolbox Basic Rel. 1.15 software.

The experimental EMC/a_w_ data were used to construct isotherm curves in Kaleidagraph for version 4.54 software [20], with a_w_ and EMC data entered onto the x- and the y-axis, respectively. The EMC equations in Table 8 were used to fit the measured moisture isotherms of polydextrose.

In this study, to investigate the effect of temperature on moisture isotherms, we suppose that EMC is the function of temperature and relative humidity, and develop a seven-parameter polynomial,

(1) $$M=A+B\cdot t+C\cdot t^{2}+D\cdot t^{3}+E\cdot a_{w}+F\cdot a_{w}^{2}+G\cdot a_{w}^{3},$$

where *M* is equilibrium moisture (%), *a*_w_ is water activity (decimal), *t* is temperature (°C), and A~G are parameters.

Fitting was conducted by nonlinear regression analysis in SPSS v17.0 for Windows (SPSS Inc., Chicago, IL, USA, [27]). The criteria used to determine the equation for the EMC/ERH data were the determination coefficient (*R*^2^), residue sum of squares (*RSS*), and standard error (*SE*), as well as mean relative percentage error (*MRE*). Equations (2)–(5) were used to calculate *R*^2^, *RSS*, *SE*, and *MRE*, respectively.

(2) $$R^{2}=1-\sum_{i=1}^{n}{\left(m_{i}-m_{pi}\right)}^{2}/\sum_{i=1}^{n}{\left(m_{i}-m_{mi}\right)}^{2}$$

(3) $$RSS=\sum_{i=1}^{n}{\left(m_{i}-m_{pi}\right)}^{2}$$

(4) $$SE=\sqrt{\sum_{i=1}^{n}{\left(m_{i}-m_{pi}\right)}^{2}/\left(n-1\right)}$$

(5) $$MRE\%=\frac{100}{n}\sum_{i=1}^{n}\left|\frac{m_{i}-m_{pi}}{m_{i}}\right|$$

where *m*_i_ is the experimental value, *m*_pi_ is the predicted value, *m*_mi_ is the average of experimental values, and *n* is the number of observations. The determinationcoefficient was one of the primary criteria for selecting the bestequation to fit the experimental data. The otherstatistical parameters, *MRE* as a percentage, *RSS,* and *SE*, were also usedto determine the quality of the fit. The fit of an equation to the EMC/ERH data was considered satisfactory if the *MRE* was lower than 10% [28].

Hysteresis degree (Hy) between adsorption and desorption is determined as:

(6) $$Hy\left(\%\right)=\frac{{EMC}_{des}-{EMC}_{ads}}{{EMC}_{ads}}\times 100$$

### 4.3. The Addition of Polydextrose in Rice Starch

The p1 polydextrose was added to rice starch with a w/w proportion of 0%, 3%, 5%, 7%, and 10%. The polydextrose-added rice starch samples were used to determine the moisture sorption isotherms of 20–35 °C using the above method. Gelatinization temperatures of the rice starch samples were determined with a differential scanning calorimeter [29]. The sample (5.0–5.1 mg) was weighed into an aluminum crucible, and deionized water was added to give a water/sample ratio of 2:1. The aluminum crucible was immediately sealed and then equilibrated overnight at 4 °C. The DSC temperature was increased from 20 °C to 110 °C at a heating rate of 10 °C min^−1^.

### 4.4. Data Analysis

Except for parallel samples for the determination of EMC/*a*_w_ data, three replicates were tested on each rice starch sample for physicochemical parameters. SPSS software (Version 17.0, SPSS Inc., [27]) was used for data analysis. One-way analysis of variance (ANOVA) and Duncan’s new multiple-range test were used to compare multiple and pairs of means, respectively. Statistical significance was declared at *p* < 0.05.

## Figures and Tables

**Table 1:** The fitting results for the moisture adsorption isotherms of polydextrose.

Equation	Sample	Equation Parameters				Statistical Parameters			
		a	b	c	d	*RSS*	*SE*	*R* ^2^	*MRE* (%)
Ferro	p1	1.244	2.841	0.552		83.1454	2.5196	0.9901	11.3825
-Fontan	p2	1.119	4.403	0.758		92.1269	2.7917	0.9887	9.2898
	p3	1.099	4.885	0.814		61.4220	1.8613	0.9917	8.8459
	p4	1.109	5.908	0.853		76.7037	2.3244	0.9894	8.3734
	p5	1.108	5.518	0.835		97.5545	2.9565	0.9867	9.8290
GDW	p1	0.672	−6.376 × 10^16^	0.934	−9.809	87.7472	2.7421	0.9895	12.4027
	p2	2.241	3.603 × 10^16^	0.941	−9.311	94.4429	2.9513	0.9885	8.9501
	p3	2.633	3.076 × 10^16^	0.942	−8.789	62.4652	1.9521	0.9916	8.3691
	p4	3.185	−4.221 × 10^16^	0.926	−9.623	75.7441	2.3670	0.9896	7.7474
	p5	3.097	5.885 × 10^16^	0.933	−9.287	96.6885	3.0215	0.9869	8.6202
Boquet	p1	1.384 × 10^−1^	−1.927 × 10^−1^	−4.296 × 10^−2^		85.1817	2.5813	0.9898	11.3607
	p2	6.955 × 10^−2^	−9.27 × 10^−3^	5.449 × 10^−2^		99.8686	3.0263	0.9878	10.7312
	p3	5.971 × 10^−2^	2.001 × 10^−2^	7.405 × 10^−2^		71.0006	2.1515	0.9904	10.5478
	p4	4.544 × 10^−2^	−9.275 × 10^−3^	5.449 × 10^−2^		91.1424	2.7619	0.9874	11.1783
	p5	4.983 × 10^−2^	3.389 × 10^−2^	7.733 × 10^−2^		116.6156	3.5338	0.9842	13.0292
Lewicki	p1	38.691	0.399	−0.252		91.4794	2.7721	0.9891	11.6048
	p2	15.802	0.626	0.195		105.9026	3.2092	0.9871	11.1774
	p3	12.847	0.672	0.457		77.4541	2.3471	0.9896	11.5859
	p4	14.923	0.613	0.556		100.4813	3.0449	0.9861	12.3879
	p5	15.121	0.615	0.446		126.6168	3.8368	0.9828	14.0529
Iglesias	p1	5.828	2.111			233.8069	6.8767	0.9719	39.7798
	p2	5.775	3.442			214.713	6.3151	0.9737	23.6655
	p3	5.502	3.686			167.7633	4.9321	0.9774	19.8521
	p4	5.424	4.706			226.4611	6.6606	0.9688	19.2150
	p5	5.464	4.432			230.5623	6.7812	0.9687	17.1287
MGAB	p1	11.146	0.924	19.784		56.4852	1.7117	0.9932	15.3594
	p2	8.848	0.945	39.663		79.2495	2.4015	0.9903	15.0906
	p3	7.585	0.957	59.231		62.9130	1.9064	0.9915	13.1556
	p4	8.481	0.938	61.969		73.5209	2.2279	0.9899	13.4585
	p5	9.211	0.931	44.778		88.1195	2.6703	0.9881	16.5624
Peleg	p1	20.311	72.472	1.416	7.227	76.1193	2.3787	0.9909	12.7732
	p2	18.333	77.065	1.027	7.213	82.8187	2.5881	0.9899	11.5972
	p3	17.251	74.145	0.947	7.166	56.1971	1.7562	0.9924	11.2992
	p4	15.861	68.392	0.7668	6.221	76.9654	2.4052	0.9894	10.7701
	p5	13.249	70.388	0.6474	5.875	92.1769	2.8805	0.9875	10.6562

Note: GDW, generalized D’Arcy and Watt; MGAB, modified Guggenheim–Anderson–de Boer; p1 to p5 are sample numbers; a, b, c, and d are the equation parameters;* R*^2^, determination coefficient; *RSS*, residue sum of squares; *SE*, standard error; *MRE*, mean relative percentage error.

**Table 2:** The fitting results for the moisture desorption isotherms of polydextrose.

Equation	Sample	Equation	Parameters			Statistical	Parameters		
		a	b	c	d	*RSS*	*SE*	*R* ^2^	*MRE* (%)
Ferro-	p1	0.939	729.174	2.474		271.7619	6.2349	0.9454	11.7105
Fontan	p2	0.936	1185.896	2.599		269.6140	8.1701	0.9466	11.2267
	p3	0.938	1130.646	2.601		264.5025	8.0152	0.9432	11.8144
	p4	0.935	1500.841	2.699		290.6119	8.8064	0.9365	12.3165
	p5	0.936	917.159	2.559		271.8086	8.2366	0.9428	11.8704
GDW	p1	12.538	−6.452 × 10^17^	0.985	−5.126	184.7613	5.7738	0.9628	9.1302
	p2	13.377	−7.379 × 10^17^	0.995	−5.161	178.9945	5.5936	0.9645	8.7242
	p3	13.095	−4.461 × 10^17^	0.981	−5.111	178.6485	5.5827	0.9617	9.4301
	p4	13.346	−1.765 × 10^18^	0.986	−4.849	205.6944	6.4279	0.9551	10.2768
	p5	12.661	3.323 × 10^18^	0.987	−4.914	188.0357	5.8761	0.9604	9.6024
Boquet	p1	−5.186 × 10^-3^	0.135	0.122		187.7547	5.6895	0.9623	9.6116
	p2	−4.957 × 10^-3^	0.128	0.115		189.0566	5.7289	0.9625	9.3613
	p3	−4.957 × 10^-3^	0.129	0.116		185.2875	5.6148	0.9602	9.8677
	p4	−5.073 × 10^-3^	0.129	0.116		222.9486	6.7561	0.9513	11.1029
	p5	−5.287 × 10^-3^	0.136	0.122		196.6704	5.9597	0.9586	10.2154
Lewicki	p1	10.293	0.694	866.000		214.2943	6.4938	0.9569	10.4041
	p2	11.002	0.674	866.000		213.6649	6.4747	0.9576	10.0556
	p3	10.844	0.664	866.000		208.2569	6.3108	0.9553	10.4865
	p4	10.925	0.658	866.000		241.9401	7.3315	0.9471	11.5011
	p5	10.323	0.685	866.000		220.6965	6.6877	0.9536	10.8351
Iglesias	p1	4.474	11.995			191.4131	5.6298	0.9615	8.8098
	p2	4.509	12.834			185.6057	5.4589	0.9632	8.4282
	p3	4.319	12.644			188.1148	5.5328	0.9596	9.1332
	p4	4.272	12.752			210.9327	6.2039	0.9539	9.9994
	p5	4.368	12.046			192.7602	5.6694	0.9594	9.3108
MGAB	p1	8.841	0.915	9.553 × 10^9^		290.0764	8.7902	0.9417	12.8749
	p2	9.407	0.906	1.067 × 10^10^		306.6459	9.2923	0.9392	12.7336
	p3	9.269	0.901	8.621 × 10^9^		293.5406	8.8952	0.9369	13.0781
	p4	9.311	0.899	1.146 × 10^10^		339.8126	10.2974	0.9257	13.7825
	p5	8.841	0.911	9.154 × 10^9^		303.4783	9.1963	0.9362	13.1996
Peleg	p1	14.803	69.557	4.795 × 10^−2^	6.175	142.7679	4.4615	0.9713	7.1544
	p2	15.649	70.007	4.615 × 10^−2^	6.162	137.3941	4.2936	0.9728	7.3192
	p3	15.185	66.077	4.065 × 10^−2^	6.013	136.8964	4.2781	0.9706	8.1769
	p4	15.506	66.721	4.609 × 10^−2^	6.226	169.0143	5.2817	0.9631	8.7802
	p5	14.831	68.398	4.708 × 10^−2^	6.238	149.8277	4.6821	0.9685	8.4051

Note: GDW, generalized D’Arcy and Watt; MGAB, modified Guggenheim–Anderson–de Boer; p1 to p5 are sample numbers;a, b, c, and d are the equation parameters;* R*^2^, determination coefficient; *RSS*, residue sum of squares; *SE*, standard error; *MRE*, mean relative percentage error.

**Table 3:** The fitting results of our developed polynomial on moisture desorption isotherms of polydextrose.

Type	Sample	Equation	Parameters						Statistical	Parameters
		A	B	C	D	E	F	G	*R* ^2^	*MRE* (%)
Ads	p1	6.485	−0.958	2.071 × 10^−2^	−1.180 × 10^−4^	84.302	−229.706	228.285	0.9866	33.7978
	p2	−10.394	1.192	−6.541 × 10^−2^	9.960 × 10^−4^	92.916	−249.788	240.707	0.9848	22.7372
	p3	−71.023	7.841	−2.974 × 10^−1^	3.640 × 10^−3^	83.347	−227.405	223.018	0.9858	19.7183
	p4	−9.024	1.397	−7.000 × 10^−2^	1.010 × 10^−3^	66.456	−183.069	191.313	0.9867	14.8199
	p5	27.979	−2.822	8.911 × 10^−2^	−9.540 × 10^−4^	65.568	−186.908	196.687	0.9862	14.9129
	Aver-ads	−13.251	1.539	−7.190 × 10^−2^	9.990 × 10^−4^	79.082	−217.042	217.289	0.9865	19.0038
Des	p1	99.812	−9.624	3.376 × 10^−1^	−3.990 × 10^−3^	69.7483	−221.873	218.486	0.9907	4.5319
	p2	65.482	−5.537	1.832 × 10^−1^	−2.090 × 10^−3^	70.029	−222.948	219.752	0.9918	4.2282
	p3	62.348	−5.044	1.608 × 10^−1^	−1.780 × 10^−3^	64.764	−207.252	206.103	0.9941	3.8077
	p4	104.235	−9.905	3.454 × 10^−1^	−4.080 × 10^−3^	67.713	−214.584	210.458	0.9927	3.9853
	p5	59.269	−5.022	1.688 × 10^−1^	−1.980 × 10^−3^	69.376	−220.301	215.993	0.9907	4.4116
	Aver-des	77.473	−6.946	2.360 × 10^−1^	−2.750 × 10^−3^	68.274	−217.403	214.324	0.9925	4.1292
	Mean	32.109	−2.703	8.220 × 10^−2^	−8.760 × 10^−4^	73.678	−217.223	215.807	0.9897	7.7274

Note: Ads, adsorption; Des, desorption; p1 to p5 are sample numbers; Aver-ads is the average of adsorption data; Aver-des is the average of desorption data; Mean is the average of adsorption and desorption data; A, B, C, D, E, F, and G are the equation parameters;* R*^2^, determination coefficient; *MRE*, mean relative percentage error.

**Table 4:** Determination of the best-fitting equations for polydextrose.

Types	Equation	Statistical	Parameters			Order
		*RSS*	*SE*	*R* ^2^	*MRE* (%)	
Adsorption	Ferro-Fontan	82.1905 ± 14.1148	2.4907 ± 0.4278	0.9893 ± 0.0018	9.5441 ± 1.1601	2
	GDW	83.4176 ± 14.2680	2.6068 ± 0.4458	0.9892 ± 0.0017	9.2179 ± 1.8340	1
	Boquet	92.7618 ± 16.9791	2.8110 ± 0.5145	0.9879 ± 0.0024	11.3694 ± 0.9841	5
	Lewicki	100.3868 ± 18.1913	3.0420 ± 0.5512	0.9869 ± 0.0027	12.1618 ± 1.1444	6
	Iglesias	214.6613 ± 27.1940	6.3131 ± 0.8007	0.9721 ± 0.0036	23.9282 ± 9.1707	8
	MGAB	72.0576 ± 12.6291	2.1836 ± 0.3827	0.9906 ± 0.0019	14.7253 ± 1.4124	4
	Peleg	76.8555 ± 13.2063	2.4017 ± 0.4127	0.9900 ± 0.0018	11.4192 ± 0.8490	3
	Polynomial	107.8395 ± 10.6417	3.7186 ± 0.3669	0.9860 ± 0.0008	21.1972 ± 7.8022	7
Desorption	Ferro-Fontan	273.6598 ± 9.9326	7.8926 ± 0.9738	0.9429 ± 0.0039	11.7877 ± 0.3898	7
	GDW	187.2269 ± 11.0583	5.8508 ± 0.3456	0.9609 ± 0.0036	9.4327 ± 0.5777	4
	Boquet	196.3436 ± 15.4681	5.9498 ± 0.4688	0.9589 ± 0.0046	10.0318 ± 0.6771	5
	Lewicki	219.7705 ± 13.1546	6.6597 ± 0.3986	0.9541 ± 0.0042	10.6565 ± 0.5475	6
	Iglesias	193.7653 ± 9.9971	5.6989 ± 0.2940	0.9595 ± 0.0035	9.1363 ± 0.5881	3
	MGAB	306.7108 ± 19.7252	9.2943 ± 0.5977	0.9359 ± 0.0061	13.1337 ± 0.4048	8
	Peleg	147.1801 ± 13.2734	4.5994 ± 0.4148	0.9693 ± 0.0038	7.9672 ± 0.7031	2
	Polynomial	38.5874 ± 8.0125	1.3306 ± 0.2763	0.9920 ± 0.0014	4.1929 ± 0.2981	1

Note: GDW, generalized D’Arcy and Watt; MGAB, modified Guggenheim–Anderson–de Boer; *R*^2^, determination coefficient; *RSS*, residue sum of squares; *SE*, standard error; *MRE*, mean relative percentage error.

**Table 5:** The parameters for the optimal equations.

Equation	Types	Equation	Parameters			Statistical	Parameters
		a	b	c	d	*R* ^2^	*MRE* (%)
Ferro-Fontan	Ads	1.125	4.501	0.763		0.9905	8.6309
	Des	0.937	1052.959	2.584		0.9438	11.7343
	Mean	0.982	38.377	1.544		0.9779	9.4624
GDW	Ads	1.376	1.090 × 10^12^	0.936	6.764	0.9902	8.3919
	Des	11.997	9.200 × 10^11^	0.985	0.421	0.9618	9.3906
	Mean	6.749	8.550 × 10^7^	0.961	1.036	0.9836	7.1238
Boquet	Ads	6.820 × 10^−2^	−6.640 × 10^−3^	5.520 × 10^−2^		0.9891	11.0751
	Des	−5.100 × 10^−3^	0.132	0.118		0.9601	9.9857
	Mean	−1.300 × 10^−3^	0.151	0.144		0.9838	6.6885
Peleg	Ads	16.837	73.032	0.944	6.766	0.9911	10.9682
	Des	15.166	68.252	4.423 × 10^−2^	6.148	0.9704	7.9234
	Mean	13.241	71.532	0.2304	6.026	0.9861	7.0115

Note: GDW, generalized D’Arcy and Watt; Ads, adsorption; Des, desorption; mean is the average of desorption and adsorption isotherms; a, b, c, and d are the equation parameters;* R*^2^, determination coefficient; *RSS*, residue sum of squares; *SE*, standard error; *MRE*, mean relative percentage error.

**Table 6:** Effect of polydextrose addition on the gelatinization properties of rich starch.

Sample	Δ*H* (J/g)	*T*_o_ (°C)	*T*_p_ (°C)	*T*_c_ (°C)	Peak Width (°C)	Peak Height (0.01 mW/mg)
CK	9.63 ± 0.03 ^a^	64.00 ± 0.10 ^a^	70.63 ± 0.31 ^ab^	78.50 ± 0.44 ^a^	7.70 ± 0.20 ^a^	16.37 ± 0.53 ^a^
3%PD	9.26 ± 0.29 ^b^	64.00 ± 0.20 ^a^	70.47 ± 0.15 ^b^	77.30 ± 0.17 ^b^	7.57 ± 0.12 ^a^	16.2 ± 0.43 ^a^
5%PD	8.91 ± 0.26 ^bc^	64.07 ± 0.23 ^a^	70.37 ± 0.15 ^b^	77.20 ± 0.46 ^b^	7.53 ± 0.21 ^a^	15.6 ± 0.95 ^a^
7%PD	8.82 ± 0.09 ^c^	64.15 ± 0.21 ^a^	70.90 ± 0.14 ^a^	77.55 ± 0.35 ^b^	7.50 ± 0.28 ^ab^	15.3 ± 0.55 ^a^
10%PD	8.81 ± 0.39 ^bc^	64.10 ± 0.20 ^a^	71.07 ± 0.32 ^a^	77.47 ± 0.35 ^b^	7.23 ± 0.06 ^b^	15.8 ± 0.44 ^a^

Note: CK, the control rice starch, PD, polydextrose; Δ*H*, enthalpy of gelatinization; *T*_o_, the onset temperature of gelatinization; *T*_p_, the peak temperature of gelatinization; *T*_c_, the conclusion temperature of gelatinization. Data are expressed as mean ± standard deviation (SD), number of repetitions *n* = 3. Means with the different superscript letters in a column are different significantly (*p* < 0.05) among different polydextrose added samples.

**Table 7:** The polydextrose samples used in this study.

No	Producing Plant	Region	Moisture Content (% Wet Basis)
p1	Renliang Biotechnology Co., LTD.	Shanghai, China	1.00
p2	Taili Jie Biotechnology Co., LTD.	Mengzhou, Henan province, China	2.60
p3	Baoling Bao Biotechnology Co., LTD.	Yucheng, Shandong province, China	3.31
p4	BailongChuangyuan Biotechnology Co., LTD.	Yucheng, Shandong province, China	4.42
p5	XingguangShouchuang Biotechnology Co., LTD.	Dezhou, Shandong province, China	4.66

**Table 8:** EMC equations used in this study.

Equation Name	Formula	Reference
Ferro-Fontan	$M={\left[\frac{b}{ln\left(\frac{a}{a_{w}}\right)}\right]}^{1/c}$	Saberi, et al. [21]
GDW	$M=\frac{a\cdot b\cdot a_{w}}{1+b\cdot a_{w}}\cdot \frac{1-c\cdot \left(1-d\right)\cdot a_{w}}{1-c\cdot a_{w}}$	Furmaniak, et al. [22]
Boquet	$M=\frac{a_{w}}{a+b\cdot a_{w}+c\cdot a_{w}^{2}}$	Boquet, et al. [23]
Lewicki	$M=\frac{a}{{\left(1-a_{w}\right)}^{b}}-\frac{a}{{\left(1+a_{w}\right)}^{c}}$	Saberi, et al. [21]
Iglesias–Chirife	$M=a\left(\frac{a_{w}}{1-a_{w}}\right)+b$	Iglesias and Chirife [24]
MGAB	$M=\frac{a\cdot b\cdot \left(c/t\right)\cdot a_{w}}{(1-b\cdot a_{w})\left(1-b\cdot a_{w}+b\cdot \left(c/t\right)\cdot a_{w}\right)}$	Cao, et al. [25]
Peleg	$M=a\cdot {a_{w}}^{c}+b\cdot {a_{w}}^{d}$	Peleg [26]

Note: GDW, generalized D’Arcy and Watt; MGAB, modified Guggenheim–Anderson–de Boer; *M* is equilibrium moisture (%), *a*_w_ is water activity, *t* is temperature (°C). a, b, c, and d are the equation parameters.

## Data Availability

The original contributions presented in the study are included in thearticle; further inquiries can be directed to the corresponding author.
